# When less is more: coy display behaviours and the temporal dynamics of animal courtship

**DOI:** 10.1098/rspb.2023.1684

**Published:** 2023-10-04

**Authors:** Thomas MacGillavry, Giovanni Spezie, Leonida Fusani

**Affiliations:** ^1^ Konrad Lorenz Institute of Ethology, University of Veterinary Medicine, Vienna, Austria; ^2^ Department of Behavioural and Cognitive Biology, University of Vienna, Vienna, Austria

**Keywords:** courtship, sexual coercion, sexual autonomy, temporal dynamics, curiosity, perceptual bias

## Abstract

Sexual selection research has been dominated by the notion that mate choice selects for the most vigorous displays that best reflect the quality of the courter. However, courtship displays are often temporally structured, containing different elements with varying degrees of intensity and conspicuousness. For example, highly intense movements are often coupled with more subtle components such as static postures or hiding displays. Here, we refer to such subtle display traits as ‘coy’, as they involve the withholding of information about maximal display capabilities. We examine the role of intensity variation within temporally dynamic displays, and discuss three hypotheses for the evolution of coy courtship behaviours. We first review the threat reduction hypothesis, which points to sexual coercion and sexual autonomy as important facets of sexual selection. We then suggest that variation in display magnitude exploits pre-existing perceptual biases for temporal contrast. Lastly, we propose that information withholding may leverage receivers' predispositions for filling gaps in information—the ‘curiosity bias’. Overall, our goal is to draw attention to temporal variation in display magnitude, and to advocate possible scenarios for the evolution of courtship traits that regularly occur below performance maxima. Throughout, we highlight novel directions for empirical and theoretical investigations.

## 1. Introduction

Courtship displays involve some of the most spectacular forms of animal signalling. These ‘strange antics’—as Darwin [1] called them—have long fascinated and perplexed human observers. Despite the astonishing variation in courtship motor patterns, most studies have predominantly focused on energetically expensive displays that demand high levels of physical exertion or coordination [2–5], and it is often implicitly assumed that mating traits will be displayed as close as possible to performance maxima. For example, Byers *et al*. [6] proposed two categories that define motor performance during courtship, namely vigour—the ability to perform energetically expensive acts at intensity maxima—and skill—the ability to perform motor tasks that require precise activation and coordination of motor units ‘close to the limit of production possibilities' [6].

**Figure 1.**
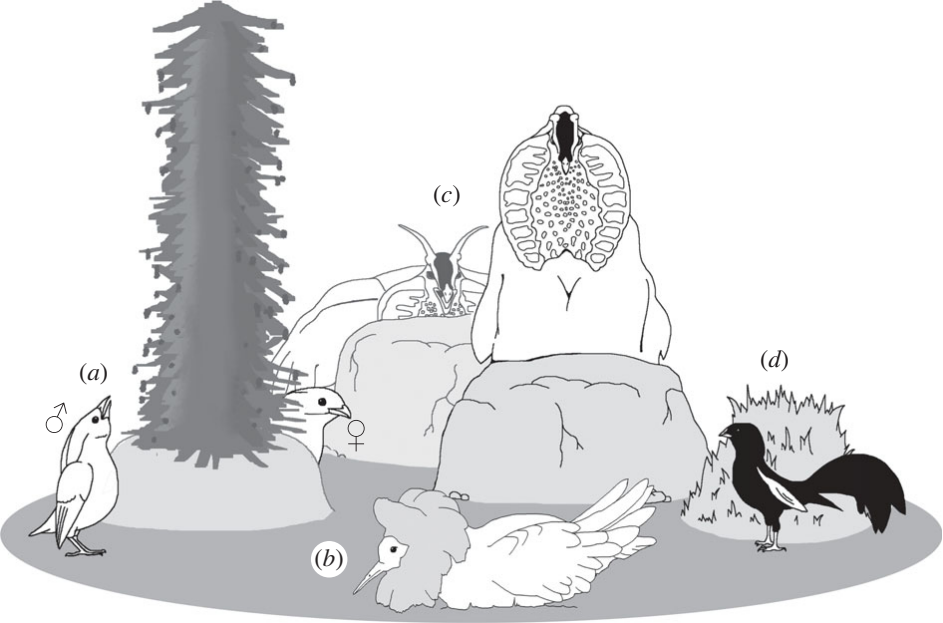
Examples of ‘coy’ display behaviours that occur below performance maxima. (*a*) Male MacGregor's bowerbirds *Amblyornis macgregoriae* perform counter-moves as the female moves around the maypole bower, thus keeping the bower between them, before flashing their brightly coloured nuchal crest [7]. (*b*) Male ruffs *Calidris pugnax* alternate vigorous chases with a static display, consisting of a motionless ‘squatting’ position [8]. (*c*) In horned pheasants (gen. *Tragopan*), males hide behind a solid object before revealing their vibrantly coloured lappet [9]. (*d*) Male Jackson's widowbirds *Euplectes jacksoni* perform counter-moves around a bower-like structure to remain hidden from an attending female [10].

However, while extravagant motor patterns are arguably more likely to capture the attention of researchers, many courtship displays are multifaceted and temporally structured choreographies, where vigorous components can vary in intensity over time, or are interspersed with more subtle elements. These include static postures or hiding displays where courters actively conceal sexual ornaments or the entire body (figure 1 and table 1). We will refer to such courtship phenotypes as ‘coy’ (see Glossary) since they involve the temporary withholding of information about maximal display capabilities. Unlike many other courtship components, coy display traits appear to be relatively inexpensive to produce, and are unlikely to act as direct indicators of vigour or skill. Rather, such traits are part of an integrated courtship display and could be interpreted as providing temporal variation in display salience or intensity. Yet, despite the widespread presence of multi-component sexual displays in animals, the role of courtship temporal dynamics in mate choice and attractiveness has seldom been explored [15–17]. In particular, a comprehensive discussion about the specific role of coy display traits is currently lacking.

**Table 1.** Different kinds of coy display behaviours. For an extended list of examples and references, see electronic supplementary material, table S1.

‘coy’ display behaviour category	example
hiding behind object or ‘bower'	crouching behind an object followed by sudden reveal in the tragopans [9]
temporary concealment of an ornament	intermittent flashing of the iridescent throat patch in the parotias [11]
reduction of display intensity	reduction of intensity in response to female position in the satin bowerbird (*Ptilonorhynchus violaceus*) [12]
static postures involving, e.g. crouching or bowing	crouching in the ruff (*Calidrix pugnax*) [8]
rotating or turning away from a receiver	rotation away from females in the Indian peafowl (*Pavo cristatus*) [13]
courter oriented backwards or approaching receivers in backwards orientation	backwards approach in bowing position in manakins (e.g. gen. *Pipra*) [14]

There are several possible reasons for the predominant emphasis on courtship performance maxima. As stated above, compared to coy displays vigorous behaviours are more likely to be noticed by investigators. Second, sexual selection research has long been dominated by the view that sex differences in mating behaviour are characterized by an undiscriminating eagerness to copulate in courters (typically males) and a discriminating passivity in choosers (typically females) [18,19]. Though this traditional view of sex roles has been questioned [20–22], the assumption that males should benefit from exhibiting persistent and energetic courtship behaviour—and that the level preferred by receivers is typically the maximum—have rarely been challenged. Finally, contemporary sexual selection research has been characterized by a particular emphasis on the ‘good genes’ model of signal evolution (electronic supplementary material, box S1), which posits that choosers evolve to prefer courtship traits that honestly signal heritable genetic quality, as this allows them to sire higher quality offspring. Though the indirect genetic benefits of mating with higher-quality males have rarely been demonstrated convincingly (see [23]), this prevailing narrative has probably resulted in a bias in studies of elaborate courtship, which often focus on vigorous and physiologically demanding motor displays, as such traits are likely the best indicators of whole-organism performance, and may therefore best signal the courter's genetic quality [6,24].

This is not to say that coy display traits—and courtship displays in general—do not evolve to signal quality. Indeed, the ability to exercise executive control over the desire to copulate, withhold information by concealing sexual ornaments, and entice receivers to approach, may be considered skillful components of courtship and are therefore potentially targets of mate choice, as cognitive traits may capture considerable genetic variation in male quality [25]. Nonetheless, many studies have focused on strenuous motor displays as they are more intuitively explained as traits that signal viability. As a result, courtship components that are characterized by low levels of showiness have largely been overlooked.

Here, we argue that to gain a complete understanding of the evolution of multi-component, dynamic courtship displays, it is also important to discuss whether and how temporal *variation* in the structure and intensity of such displays may affect mate choice. In particular, more attention should be devoted to investigating the role of courtship motor components that are displayed at submaximal intensity, or where information acquisition is deliberately prevented or delayed. We reiterate that our focus on such coy display traits does not aim at challenging the role of highly coordinated and physically demanding displays in mate choice [3,5], but rather at highlighting the scarcity of studies and hypotheses regarding the full spectrum of temporal variation in display magnitude, including subtle courtship components.

In this essay, we explore three hypotheses for the evolution of coy courtship displays. First, we discuss the hypothesis that such courtship traits reduce the potentially threatening or startling effect of vigorous displays, which suggests a role for sexual coercion in the evolution of sexual displays [26–28]. This explanation has been invoked to interpret the evolution of bowers in bowerbirds (see below), though it has rarely been investigated in other taxa and remains underappreciated in sexual selection research [26]. The ultimate cause of such traits may be an enhancement of the sexual autonomy of choosers (see Glossary), the importance of which has only recently been acknowledged [27–29].

Second, we discuss how temporal variation in display intensity may affect receivers' perceptual systems, and hence their mating preferences, by leveraging pre-existing biases for temporal contrast in visual stimuli [30]. In addition, we suggest a third hypothesis, namely that courtship components that involve the withholding or delaying of information acquisition may exploit the intrinsic rewards derived from information seeking, namely, the perceptual and cognitive mechanisms aimed at filling gaps in knowledge [31,32]. Such hiding displays may thus leverage a widespread bias for information seeking in choosers, which we refer to here as the ‘curiosity bias’.

While we specifically aim to draw attention to the coy elements of courtship displays, such behaviours are probably best explained as part of dynamic and integrated courtship *performances*. Indeed, the temporal structure of elaborate display choreographies and the way such dynamics are processed by receivers are both fruitful avenues for future research. Few studies have attempted to discuss why different components of motor displays are arranged and structured as they are. With this review, we hope to lay the groundwork for researchers to appreciate the coy as well as the vigorous components of courtship phenotypes, and to stimulate further discussion about how courtship performances unfold over time.

## 2. Sexual coercion and the evolution of variation in courtship intensity

### (a) Sexual coercion and sexual autonomy

Sexual autonomy can be defined as the degree to which individuals are able to freely select their mates, while sexual coercion is the strategy of copulating with others by directly subverting their autonomous mating decisions [28]. Sexual coercion may incur moderate to severe costs to recipients, ranging from physiological stress [33] to physical harm and even death [34,35]. Females may also experience substantial indirect costs in the form of less viable or attractive offspring [29]. Altogether, the magnitude of the costs experienced by the recipients of sexual coercion have driven the evolution of a variety of resistance mechanisms, including a general aversion to coercive courters [29]. In addition to morphological resistance mechanisms, such as the elaborate genitalia of female waterfowl [36,37], behavioural resistance mechanisms may have far-reaching effects on a species's social dynamics during courtship interactions.

For example, in the Japanese quail *Coturnix japonica*, females avoid male sexual harassment by forming coalitions [38], shifting daily mating patterns [39], affiliating with less aggressive males [40], and even prefer to affiliate with the losers rather than winners of agonistic interactions [41]. Together with studies across a wide variety of animal taxa including arthropods [42], squamates [43], birds [35] and mammals [34], this suggests that there is strong selection for females to resist sexual coercion or avoid physical harm inflicted by coercive males [29,34,35]. In particular, mate choice may drive the evolution of courtship components that reduce the perceived risk of sexual coercion. However, sexual coercion is often overlooked as a selective force in studies of animal behaviour [29], and the role of sexual autonomy in mate choice has rarely been discussed [28].

### (b) The threat reduction hypothesis

**Figure 2.**
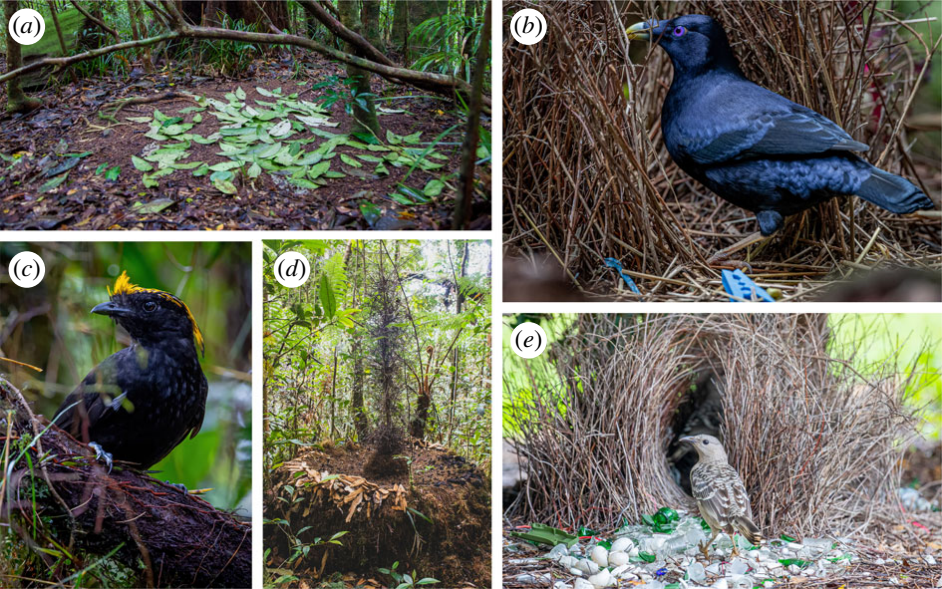
(*a*) The court of a tooth-billed bowerbird *Scenopoeetes dentirostris*. (*b*) A male satin bowerbird *Ptilonorhynchus violaceus*. (*c*) A male Archbold's bowerbird *Archboldia papuensis*. (*d*) The bower of MacGregor's bowerbird. (*e*) A male great bowerbird *Chlamydera nuchalis* at its bower. Photos by Dominic Chaplin.

While sexual coercion is relevant to the evolution of mating behaviour in all species exhibiting some form of mate choice, its importance in the evolution of courtship phenotypes has been studied almost exclusively in bowerbirds, which build ornate structures called bowers (figure 2).

Borgia [44] initially proposed the threat reduction hypothesis to explain the function of these structures, which posits that bowers reduce the threatening effect of vigorous displays by providing females with a protective space to view courtship, thereby facilitating escape from forced copulation attempts [26,44]. While the effects of bowers on the chromatic adaptation and perspective of receivers indicates that their evolution was influenced by a variety of other factors [45,46], this hypothesis suggests that bowers first originated to reduce the threat levels associated with the risk of sexual coercion in receivers [26].

Overall, threat reduction also represents a plausible explanation for the evolution of coy courtship behaviours across the animal phylogeny. Since courtship often involves close proximity interactions between the sexes—probably to closely evaluate the phenotypes of prospective mates—there is also an enhanced risk of sexual coercion for females. It may then be a general feature of animal courtship that extremely vigorous displays are also more threatening to receivers as they resemble aggressive attempts at forced copulation. The threat reduction hypothesis may therefore explain many other coy courtship phenotypes, especially in taxa where sexual coercion is common (e.g. Galliformes). For example, copulation in the Indian peafowl *Pavo cristatus* occurs after the courting male rushes towards a female (the ‘hoot-dash’ [13]). The ‘coy’ phase of the display, where the male turns away from the female before the hoot-dash [47], may therefore function to reduce receiver threat levels and prevent startling.

Indeed, temporal variation in display intensity may have evolved to offset the threatening effect of vigorous components of courtship. This may especially be the case when vigorous display elements resemble attempts at forced copulation, such as the ‘mock-attack’ of the spotted bowerbird *Chlamydera maculata* [48] or the aforementioned ‘hoot-dash’ of the Indian peafowl [13]. However, at least in bowerbirds, when females are not startled, they tend to prefer more intense displays [12]. While mate choice may select for vigorous, and even aggressive vocal and motor displays, such performances only become threatening when they exceed a certain intensity or are produced at inappropriate times. Courters may then be selected to flexibly modulate the intensity of their displays, or evolve courtship components that are inherently coy, such as hiding displays. Thus, by punctuating otherwise vigorous courtship movements with coy display elements, courters may enhance their mating success by mitigating the probability of a prospective mate terminating courtship [12,48].

Furthermore, males may also benefit by concealing traits that may facilitate coercion, such as weapons, as they may have a threatening effect, though this has not been tested. Similarly to Pradhan & Van Schaik's [49] coercion mitigation hypothesis—which predicts that choosers evolve not to prefer weapons as they can be used for coercion—the threat reduction hypothesis predicts that courtship often involves behaviours that reduce the salience of weapons and other threatening stimuli, especially when there is little sexual conflict over the expression of weapons in courters (e.g. when intrasexual competition is low).

Females, on the other hand, may also prefer larger weapons either because this enhances the competitiveness of male offspring or because weapons may also serve as sexual signals [50,51]. Mate choice and intrasexual competition may thus generally act in tandem, resulting in a covariance between the magnitude of ornaments (including displays) and weapons [52]. If those weapons and other traits are used for coercion, then the evolution of animal courtship results from a complex interaction between intrasexual competition, mate choice and sexual coercion. We predict that coy courtship elements with threat-reducing functions—where the salience of weapons and aggressive motor patterns is either lowered or modulated—are most common in species where sexual coercion is frequent during courtship interactions.

While the initial iteration of the threat reduction hypothesis seems intuitive, further exploration suggests that it is more theoretically complex than previously anticipated. At least three hypotheses which may explain coy display behaviour are nested within the general model: (a) coy traits evolve to avoid triggering a pre-existing, automatic startle response in receivers, (b) coy traits evolve to avoid threatening or startling receivers, which occurs to avoid the direct costs of sexual coercion, and (c) that coy traits evolve to avoid threatening or startling receivers, which occurs to avoid the *indirect* cost of sexual coercion in the form of less attractive offspring.

This third, most elaborate version of the threat reduction hypothesis has recently been explored using quantitative mathematical models by Snow & Prum [53]. Their proof-of-concept analysis suggests that, given a specific level of coercion, male traits that enhance female sexual autonomy—which they call ‘autonomy-enhancing’ traits—may indeed persist in the population as females experience indirect benefits from mating with more attractive males. When the efficiency of protection against coercion incrementally increases, this theoretical process—dubbed evolutionary ‘remodelling’—may then gradually remove sexual coercion from the population.

While this model can only be applied to coy displays that also enhance the sexual autonomy of receivers, the general framework of the threat reduction hypothesis covers more ground in explaining coy behaviours that do not necessarily prevent sexual coercion. In the following section, we review the existing empirical evidence for the threat reduction hypothesis, and discuss some of its shortcomings.

### (c) Evidence and possible criticisms

The first studies to provide support for the threat reduction hypothesis focused on the spotted bowerbird, which produces some of the most vigorous displays among bowerbirds. Bowers in this species are avenues made of sticks and dry grasses, allowing receivers to peer through them, thus creating a direct barrier between the courter and receiver [44]. In an experiment where one of the bower's walls was destroyed, females oriented themselves more towards the intact bower wall during courtship [54], in line with the prediction that females feel less threatened when observing courtship from behind a protective barrier. This ability to modulate the intensity of display has further been demonstrated in male satin bowerbirds with experiments using robotic models of females [12,55].

Courtship in many bird species appears to be punctuated by low-intensity enticement phases, such as hiding, followed by high-intensity chases. However, since females persistently return to the court despite being repeatedly chased, it remains unclear whether this behaviour is an attempt at copulation, or rather represents a vigorous courtship element that has been misinterpreted as attempted copulation by human observers. Conversely, it is possible that many courtship behaviours across animal species actually represent forms of sexual coercion and have been misinterpreted as vigorous forms of display.

In summary, mate choice may act to maximize the sexual autonomy of choosers since those individuals that are better able to evaluate and freely select their mates benefit both directly and indirectly from reproducing with more attractive individuals [53]. Given the direct, often physical costs of sexual coercion, this prediction holds true for all relevant models of sexual selection (Fisherian runaway, indicator mechanisms, perceptual biases, and chase-away selection [56]). The threat reduction hypothesis thus predicts that courters may evolve displays that indicate that they will not coerce a visiting female during courtship, namely coy display phenotypes.

While the threat reduction hypothesis is a promising explanation for the evolution of coy courtship phenotypes, more research is needed across diverse taxa to determine the importance of sexual autonomy and sexual coercion in the evolution of courtship displays. An outstanding question in the threat reduction hypothesis is *why* females may startle in response to vigorous courtship. There is a notable lack of alternative hypotheses to explain this phenomenon, and previous studies implicitly assume that females startle and escape due to the perceived risk of sexual coercion. One alternative explanation may be that both sexes automatically startle in response to vigorous stimuli of any kind, for example due to responses linked to anti-predatory behaviour. For instance, in spotted bowerbirds, both male as well as female bower-visitors appear to be startled by the vigorous displays of dominant males (G.S. 2018–2019, personal observation). Threat reduction may therefore have less to do with evolutionary ‘remodelling’ [53] than simply reducing the risk that receivers terminate courtship because of a reflexive, pre-existing startle response. Also, rather than only being threatening, connoting a negative valence, vigorous courtship elements may have a positive, stimulating effect on receivers. Such an effect may be due to predispositions in receivers for specific temporal features of sexual displays. We elaborate on this hypothesis in the next section.

## 3. The role of receiver biases in the evolution of temporally dynamic displays

Sensory biases in sexual communication have been a popular theme for over 30 years [30,57–61]. Sensory bias models posit that pre-existing properties of sensory systems that evolved in specific contexts under natural selection can affect preference in different communication contexts, namely sexual signalling [60]. One example is sexual ornaments whose colour, shape or movement trigger pathways that originally functioned in food detection (e.g. [62,63]).

In this section, we discuss the role of pre-existing receiver biases in the evolution of sexual displays with complex temporal dynamics. First, we discuss the hypothesis that temporally structured displays may affect the attentional state of receivers by enhancing perceptual contrast between consecutive display elements (the temporal contrast hypothesis). Second, we propose that hiding displays that prevent receivers from acquiring immediate or unambiguous information about sexual signals may leverage taxonomically widespread predispositions for information seeking. We refer to these proclivities as the *curiosity bias* hypothesis.

### (a) Receiver biases for temporal contrast

The role of contrast in signal efficacy has been primarily investigated in static visual signals in terms of chromatic and brightness contrast. A large body of evidence shows that (sexual) stimuli that better contrast against the background are often preferred [64–66], plausibly because these signals are easier to detect and remember [30], and metabolically cheaper to assess [67]. In comparison, fewer studies have investigated how temporal contrast (modulation of signal properties over time) in dynamic displays may impact the attractiveness of visual signals. We argue here that variability in display structure and intensity over time may enhance perceptual contrast between consecutive display elements through changes in movement patterns, thus maximizing attraction and stimulation in receivers (temporal contrast hypothesis). Similarly to static visual signals [68], the magnitude of temporal contrast would guide receivers' attention, thus ensuring better access to perception. Variation in intensity may occur by means of sequences of differently intense components, or by varying the intensity of a given component over time.

Two related mechanisms are likely to underlie receiver biases for temporal contrast in dynamic visual displays: release from habituation and enhanced memorability. Habituation is an adaptive and taxonomically ubiquitous process that allows animals to reduce attention to repeated stimuli and respond selectively only to salient input from the environment [69]. Selection should therefore favour sexual signals that exploit these propensities inherent in receivers’ sensory systems. Though empirical evidence suggests that aversion from predictability and preference for signal novelty are prevalent across taxa [70–72], only few studies have investigated the effect of release from habituation via temporal contrast in the context of sexual signalling. One example is Hartshorne's [73] anti-monotony hypothesis to explain birdsong complexity. According to this hypothesis, females (and same sex-competitors) are more stimulated by complex songs as larger song repertoires reduce habituation to repeated stimuli. For example, a study on grackles—a species without complex song repertoires—showed that females were more attracted to artificial stimulus songs with different syllable types than to repetitions of the same syllable [74].

Second, Guilford & Dawkins [30] suggested that perceptual contrast is one of the features of signal design that foster memory and learning, typically via increased salience [68]. Experimental evidence on the relationship between visual contrast and memorability in animals is again restricted to static visual signals, and particularly abundant regarding food aversion and aposematic coloration [75,76]. One exception is Akre & Ryan's [77] study on courtship calls in Túngara frogs, which showed that female receivers have better memory of courtship calls that vary in temporal acoustic structure than simple ones. Thus, similar to acoustic signals, temporal contrast in visual dynamic displays may mitigate habituation, enhance memorability, hence ultimately influencing attention levels, stimulation, and preference.

However, simply incorporating additional components (i.e. increasing signal complexity) may be sufficient to introduce variation in temporal structure, independently of variation in intensity. So what is the role of intensity variation between (or within) courtship components, commonly observed in dynamic sexual displays? An important point here is that intensity fluctuations may have a potentiating effect on contrast, similar to *crescendo* effects in music, which would accentuate sequence transitions and further assist courters in focusing receivers' attention, ultimately increasing stimulation and/or maximizing the transfer of relevant information encoded in specific courtship components. For example, Frith & Frith [78] suggested that the ‘hiding’ display in the courtship of Tooth-billed bowerbirds evolved to accentuate their elaborate vocal displays. This hypothesis therefore posits that signals across different sensory modalities may interact with one another during multimodal displays, and that certain display components must be reduced to maximize the efficacy of others. Future studies should explicitly strive to disentangle the role of intensity modulation from complexity *per se*, and to further investigate the impact of both on receivers' sensory systems and attention levels.

In addition, some examples of coy signals described above (table 1) suggest that mechanisms other than enhancement of temporal contrast appear to be at play. The fact that courters do not only vary display structure or intensity, but often *actively* conceal their bodies or ornaments during courtship routines suggests a role of information seeking processes. Next, we discuss the hypothesis that information withholding during courtship may exploit pre-existing cognitive mechanisms that mediate information acquisition and exploratory behaviours in receivers—the *curiosity bias* hypothesis.

### (b) Receiver biases for information seeking: the ‘curiosity bias’

The definition of ‘curiosity’ varies substantially across disciplines [31,79–82], though all definitions include the drive to seek and gather information about unfamiliar stimuli. Curiosity is typically associated with a positive response to novelty and followed by the reduction in uncertainty about the environment [81]. For the purposes of our discussion, we examine both instrumental and non-instrumental forms of information seeking (see Glossary), as both forms may act as perceptual predispositions that bias preference for stimuli in other contexts.

There are many examples showing that animals invest greatly in information seeking with or without instrumental incentives across developmental stages. A propensity towards information seeking and exploration-like behaviours appears to be related to a variety of perceptual systems and behaviours under natural selection, including foraging [83], motor learning and object play [84,85], among others [32].

Furthermore, a growing body of evidence on the neurobiological underpinnings of curiosity shows that information functions as a reward in humans and other animals. Jepma *et al*. [86] showed that the relief of perceptual curiosity resulted in the increased activation of neural structures associated with reward processing in humans (see also [87–90]). Similar results were found in some non-human animals. In a study conducted on macaque monkeys, subject animals could choose between receiving advanced information about an uncertain food reward, or non-informative cues that did not reveal the outcome [91]. Monkeys more often chose the first option, even though the information obtained did not influence the likelihood of receiving a food reward. Interestingly, information about food rewards and primary rewards themselves were shown to activate the same brain regions [91,92]. Similar studies in humans [88,93,94], macaques [95], pigeons [96] and starlings [97] showed that individuals often choose to sacrifice part of a reward (food or money) to receive advanced non-instrumental information about uncertain rewards. These results support the notion that information and the release of uncertainty about the environment are intrinsically motivating (reviewed in [92,98]), even in the absence of instrumental value of such information (the information-as-reward hypothesis [99]).

Thus, curiosity—or at least the propensity towards engaging with contexts that can disclose information about the environment—is most likely widespread across taxa. Also, predispositions towards intrinsically motivated information seeking—as well as the rewarding mechanisms underpinning these processes—are plausibly adaptive and under natural selection. Here, we argue that the above processes have the potential to drive the evolution of courtship components also in a sexual context. Coy components that involve the alluring withholding (or delayed gathering) of information about prospective mates and a temporary increase in uncertainty about the world have the potential to exploit the intrinsically motivated drive towards information seeking.

Hiding displays with these characteristics are common in a number of bird species (electronic supplementary material, table S1). For example, the bowers of male bowerbirds allow them to control the perceptual experience of females and to withhold visual information during their displays. In the Vogelkop bowerbird *Amblyornis inornatus*, for example, this coyness is particularly apparent. In this species, males hide from females inside their hut-like maypole bowers from which they call to entice the female to approach. This bower design therefore appears to have evolved to allow males to conceal themselves from attending females, which appear to be enticed to approach to gather information about the courting male.

Thus, coy display traits may benefit the sender by enhancing reproductive success if they ultimately influence mate choice. But *how* is leveraging predispositions for information seeking expected to affect mate choice? What are the mechanisms by which evolution may co-opt these predispositions into a sexual context? We suggest that tactical withholding of information during courtship (and the subsequent relief of uncertainty) may (a) leverage the reward associated with information acquisition and (b) enhance signal memorability, thus affecting mating preferences.

Because relief from uncertainty activates pathways associated with reward processing [88–90], coy elements may thus exploit the reward deriving from information seeking and influence courtship success. However, it still remains unclear how the anticipation of reward and pleasure in non-reproductive contexts may affect sexual preferences. Some evidence shows that a re-organization in neural wiring or simple genetic changes can mediate modifications in the hedonic value of a signal (reviewed in [100,101]). Another hypothesis that has been suggested in the cognitive sciences is that observers may be unable to pinpoint the source of pleasure while processing information that is deemed pleasurable, and as a result of a misattribution, observers would then tend to ascribe the source of pleasure to the displaying individual and the stimulus itself, rather than on the modalities of information processing. This misattribution would therefore be expressed as a preference for such pleasurable stimuli in the absence of an evaluation of stimulus benefits [67]. While this hypothesis remains speculative, it lends itself to multiple predictions and experimental tests (see [102]).

A second possibility is that, similar to temporal contrast, information seeking may influence mating preferences by affecting the attentional state of receivers, therefore enhancing signal memorability, namely how easily the signal—and by extension, the courter—can be remembered. Thus, we argue that curiosity and the delayed acquisition of information may be additional features that make mating signals more memorable. At least in humans, there is neurophysiological evidence that the relief of perceptual curiosity results in an increased activation of hippocampal regions and enhanced memory, thus learning and memory are facilitated following reduction of uncertainty [86]. Similarly, trivia questions that elicit higher self-reported curiosity are those that are better remembered by participants [88,89,103].

As stated above, very few studies have investigated the role that signal memorability plays in mating decisions (e.g. [77]) and the plausible additive effect of relief of uncertainty remains an unexplored area of research. More experimental programmes are needed to test whether information withholding may enhance signal memorability, as well as to distinguish between the role of contrast and curiosity in enhanced memorability (electronic supplementary material, box S2). One further prediction that follows from this hypothesis is that coy courtship traits should be more widespread in species where females sample different males multiple times before choosing a partner to mate with [e.g. 104], making memorability a fundamental component of mate choice.

Overall, the evidence we reviewed in support of the curiosity bias suggests a link between informational demands and aesthetics [67,79]. In the past 10 years, great efforts have been devoted to developing multi-disciplinary approaches to investigate the regulatory mechanisms for information seeking and curiosity in humans and other animals [99]. More experimental studies are needed to determine the role of these processes in a sexual context.

### (c) Evidence and possible criticisms

Incorporating temporal variation into courtship routines and introducing uncertainty about a signal may increase both the time and costs required for signal assessment by receivers, thus potentially reducing its efficacy in the short term. Prior work suggested that selection should favour signals that also maximize signal efficiency (i.e. properties of signals that allow their assessment at low metabolic costs via information compression [67]). Several lines of evidence suggest that signals that are cheap to assess (like symmetrical or average stimuli) are indeed preferred by receivers [105,106], also in sexual contexts [107,108]. By contrast, the hypotheses discussed here suggest that delaying information transfer may also positively influence mating preferences. One possible answer to this apparent contradiction is that the importance of efficient signal assessment may depend on ecological and life-history traits. For example, rapid assessment may be important when predation risk is high, or signal assessment is time-constrained, such as in short-lived organisms, whereas in long-lived species repeated interactions might be beneficial (or necessary) to resolve mating decisions, thus allowing space for coy traits to evolve. Future meta-analyses could investigate this purported relationship between signal efficiency, mating decisions, and life-history traits such as longevity or mating system. Alternatively, if displays that vary in conspicuousness can better focus receivers' attention than invariable repeated stimuli, this may instead further increase efficiency and facilitate perceptual processing, though these hypotheses require further empirical tests.

One additional complication is that designing experiments to test the curiosity bias hypothesis in non-human animals could be challenging. To investigate curiosity in non-human receivers, researchers obviously cannot rely on self-reports and questionnaires. It is therefore necessary to measure particular behaviours involved in information seeking such as attention and exploration (electronic supplementary material, box S2). Furthermore, comparative approaches could be developed by replicating the same experimental procedures in different species, for instance by presenting perceptual tasks that allow repeatable and valid measurements in species with different perceptual systems and morphology. Finally, because the hypotheses proposed in this essay are not necessarily mutually exclusive and their predictions are likely to overlap to a certain extent, empirical tests should be carefully designed to allow researchers to disentangle the role of threat reduction from sensory biases, and in particular the role of temporal contrast from curiosity-like processes. For example, it would be informative to test the experimental animals for those perceptual and cognitive processes that are unique to information seeking—such as novelty, surprise and anticipatory processes (electronic supplementary material, box S2).

## 4. Taxonomic distribution

In our selective review of courtship behaviours, we have identified multiple examples of coy displays, particularly across the avian phylogeny, including landfowl (Galliformes), basal landbirds (Otidiformes and Charadriiformes) and diverse songbirds (Passeriformes; e.g. in the families Cotingidae, Pipridae, Ptilonorhynchidae, Paradisaeidae, Nectariniidae and Ploceidae). While the threat reduction hypothesis has thus far only been tested in bowerbirds, sexual coercion and startling behaviour are relevant to the evolution of sexual displays across all animal taxa and may thus have shaped the evolution of elaborate temporal patterns in sexual displays in diverse species [53].

Alternatively, particularly in some arthropods, coy display elements and intensity modulation may have evolved to mitigate the risk of sexual cannibalism in courters, especially in groups such as mantids (Mantodea) or spiders (Araneae) [109]. For example, male Chinese mantis *Tenodera aridifolia* approach females more carefully during courtship interactions when the risk of being cannibalized is greater [110]. Thus, when larger females are threatening to courting males, displayers may need to modulate the intensity of their courtship to reduce the risk of sexual cannibalism (e.g. [111]). While these displays are quite divergent from our examples of coy displays in birds, the elaborate courtship displays of some arthropods are nonetheless promising to study coy display elements and the temporal structure of courtship performances.

Like threat reduction, predispositions towards temporal contrast represent additional features of animal perceptual systems that may shape the evolution of courtship phenotypes and mating preferences across a wide range of taxa. In particular, investigating release from habituation in dynamic visual signals is a particularly promising avenue of future research in both vertebrate and invertebrate model systems, as the neurological mechanisms that regulate habituation to repeated stimuli have been analysed in great detail in a diverse range of model systems (e.g. [112,113]). A study in humans [114] showed that repetition suppression (defined as the attenuation in neural activity following signal repetition) also depends on whether or not receivers can predict the re-occurrence of a given stimulus, therefore suggesting that top-down processes (along with bottom-up constraints in the peripheral sensory systems) are also involved. It would be useful to investigate whether congruence and predictability of dynamic sexual displays are important facets of perceptual processing of sexual stimuli also in other animals, and the emerging use of gaze-tracking methodologies may aid researchers in testing predictions about attention and anticipation resolution (electronic supplementary material, box S2).

Regarding curiosity, information-seeking processes have been proposed by some authors to rely on meta-cognitive functions such as an awareness of the animal's own knowledge states [115], which, according to this hypothesis, makes these processes more likely to be observed in a narrower range of model systems. However, recent studies have started to reveal forms of non-instrumental exploratory behaviours also in arthropods [116] and other invertebrates [117]. It is therefore not implausible that simple forms of cognitive predispositions towards information seeking may be shared across a broad range of taxa, also in a sexual context, especially in light of the complex cognitive processes involved in mating decisions that have been identified in arthropods [118,119].

## 5. Concluding remarks

The overarching goal of this review was to challenge the notion that mate choice selects for only the most extravagant and vigorous courtship display phenotypes. We aimed to draw attention to courtship displays that vary in their temporal patterns, often exhibiting ‘coy’ components that involve the withholding of maximum display abilities. While the notion of coyness may appear provocative in reference to courtship [120], previous hypotheses have been devised to explain the evolution of coy display traits, though they have largely been overlooked. For example, Zahavi & Zahavi [121] suggested that the ‘turning away’ phase of courtship in the Indian peafowl (see above) evolved in males to allow them to gauge the sexual interest of females: if peahens do not follow the male's movements, then courtship is unlikely to result in copulation.

Our primary aim was to highlight the importance of tackling the full spectrum of variation in display magnitude in order to gain a complete understanding on the evolution of courtship behaviour in animals. Furthermore, by focusing on sexual autonomy and perceptual aspects of courtship routines, this review suggests further examples where receivers—and receiver psychology—act as the selective agents shaping signal design (electronic supplementary material, box S1). While we have touched upon two possible mechanisms—namely, the aversion to sexual coercion and pre-existing perceptual or cognitive biases—much remains to be explored about how the psychology of animals influences the evolution of mating preferences and, as a result, courtship displays [122]. For example, the temporal variation of receivers' attention levels may play a key role in the organization of different display components within courtship performances, and similar questions have the potential to inspire inter-disciplinary research involving comparative behavioural and neurological studies, also covering themes from the emerging field of neuroaesthetics [123].

Ethology has had a long history of dissecting behaviours into their constituent parts—a view that has potentially undermined the relationships between those parts and how they are perceived by receivers. Much like human music or theatre, courtship displays may constitute true performances in their own right, where different elements interact to entice, build suspense, surprise, and excite the intended audience, all the while minimizing threat levels. The way in which such performances unfold over time represents a promising and novel direction for studies of courtship behaviour and perceptual systems in receivers.

## Data Availability

The data are provided in electronic supplementary material [125].

## Glossary

Coy display traits: We refer to coy display traits as both (i) courtship elements that can vary in intensity, for example when courters tactically adjust display intensity of a given body movement (e.g. [12]), and (ii) courtship components that are coy *per se* (listed in table 1). ‘Coy’ is often treated as a synonym of ‘choosy’, but also implies a discriminating passivity in receivers [18,20,120]. Here, it refers to the withholding of display components (such as ornaments or body parts) by courters during display, as well as to the alluring nature of these signals, especially in the cases where information acquisition in the receiver is delayed.
Curiosity: Broadly defined as ‘a motivation to seek out and acquire information about something unfamiliar’ [31]. Curiosity can be intrinsically and extrinsically motivated, depending on the source of the drive for information seeking [79], and instrumental or non-instrumental, depending on the value of the information being acquired, namely whether or not it serves an immediate function. Most authors and studies limit the definition of ‘true’ curiosity to non-instrumental and intrinsically motivated forms of information seeking. However, it is not possible to always draw clear distinctions between the processes involved.
Information withholding: Tactical concealment of phenotypic features such as ornaments, behavioural displays or the whole body.
Startle response: A rapid, protective sensorimotor response, often in response to predators [124].
Sexual coercion: The ability of individuals to copulate with others by directly subverting their independent choice of mates.
Sexual autonomy: The ability of individuals to pursue mating decisions free from coercion.
